# Impact of Cardiovascular Calcifications on the Detrimental Effect of Continued Smoking on Cardiovascular Risk in Male Lung Cancer Screening Participants

**DOI:** 10.1371/journal.pone.0066484

**Published:** 2013-06-20

**Authors:** Pushpa M. Jairam, Pim A. de Jong, Willem P. T h. M. Mali, Ivana Isgum, Harry J. de Koning, Carlijn van der Aalst, Matthijs Oudkerk, Rozemarijn Vliegenthart, Yolanda van der Graaf

**Affiliations:** 1 Julius Center for Health Sciences and Primary Care, University Medical Center Utrecht, Utrecht, The Netherlands; 2 Department of Radiology, University Medical Center Utrecht, Utrecht, The Netherlands; 3 Image Sciences Institute, University Medical Center Utrecht, Utrecht, The Netherlands; 4 Department of Public Health, Erasmus Medical Center Rotterdam, Rotterdam, The Netherlands; 5 Center for Medical Imaging – North East Netherlands, University of Groningen, University Medical Center Groningen, Groningen, The Netherlands; 6 Department of Radiology, University of Groningen, University Medical Center Groningen, Groningen, The Netherlands; University of Adelaide, Australia

## Abstract

**Background:**

Current smokers have an increased cardiovascular disease (CVD) risk compared to ex-smokers due to reversible as well as irreversible effects of smoking. We investigated if current smokers remain to have an increased CVD risk compared to ex-smokers in subjects with a long and intense smoking history. We in addition studied if the effect of smoking continuation on CVD risk is independent of or modified by the presence of cardiovascular calcifications.

**Methods:**

The cohort used comprised a sample of 3559 male lung cancer screening trial participants. We conducted a case-cohort study using all CVD cases and a random sample of 10% (n = 341) from the baseline cohort (subcohort). A weighted Cox proportional hazards model was used to estimate the hazard ratios for current smoking status in relation to CVD events.

**Results:**

During a median follow-up of 2.6 years (max. 3.7 years), 263 fatal and non-fatal cardiovascular events (cases) were identified. Age, packyears and cardiovascular calcification adjusted hazard ratio of current smokers compared to former smokers was 1.33 (95% confidence interval 1.00–1.77). In additional analyses that incorporated multiplicative interaction terms, neither coronary nor aortic calcifications modified the association between smoking status and cardiovascular risk (P = 0.08).

**Conclusions:**

Current smokers have an increased CVD risk compared to former smokers even in subjects with a long and intense smoking history. Smoking exerts its hazardous effects on CVD risk by pathways partly independent of cardiovascular calcifications.

## Introduction

Cardiovascular disease (CVD) is one of the major causes of death worldwide. As many as 30% of the deaths from CVD are attributed to cigarette smoking [1], [2]. Previous studies have consistently demonstrated that smoking cessation rapidly and markedly reduces coronary heart disease (CHD) risk [3], [4]. One year after quitting, the risk of CHD decreases by 50 percent, indicating that certain mechanisms by which smoking induces CVD are reversible to some extent [5]–[7]. However, smoking also has irreversible effects, by which former smokers continue to have an elevated CVD risk for a long time, even years after they have quit smoking [8]–[10]. Subsequently, it could be hypothesized that the advantageous effect of smoking cessation on CVD risk may be attenuated in subjects with an extended and intense smoking history. So, we aim to investigate if in a population containing subjects who have a common long and intense smoking history current smokers have a different CVD risk compared to ex-smokers. Furthermore, smoking is associated with coronary and aortic calcifications. These calcifications play an important role in plaque vulnerability which is considered to cause an increased CVD risk [8]–[10]. We, in addition, intend to investigate whether the relation between current smoking status and CVD risk is independent of, or modified by, cardiovascular calcifications.

## Materials and Methods

### Study Population

This is an ancillary study of the Dutch and Belgian Lung Cancer Screening Trial (NELSON trial; ISRCTN63545820) [11]. NELSON was approved by the Dutch and Belgian Ministry of Health and by the ethical review board of the participating hospitals. Written informed consent was obtained from each participant. The trial population comprised subjects between the ages of 50 and 75 years with a smoking history of >15 cigarettes a day for >25 years or >10 cigarettes a day for >30 years, and who were current smokers, or former smokers who quit smoking <10 years ago. Exclusion criteria for participating in the lung cancer screening trial were self-reported moderate or poor health with inability to climb two flights of stairs, recent chest CT, current or previous history of cancer, and body weight ≥140 kg. At baseline, participants in the NELSON-trial filled in a questionnaire regarding their current smoking behavior and the number of pack years smoked. A pack year is defined as twenty cigarettes smoked everyday for one year. Subjects who were active smokers at the time of scanning were classified as current smokers [11].

### Sample Selection and Study Design

In the present study, 3559 male participants from two participating hospitals, University Medical Center in Groningen (UMCG) and University Medical Center in Utrecht (UMCU), represent the full cohort and were considered for analyses. We used a case-cohort design as introduced by Prentice [12], that consists of cases and a subcohort sample that is randomly sampled from the full cohort at the beginning of the study. Subjects who developed a cardiovascular event during follow-up were identified as cases. We selected a random sample of ≈ 10% (n = 341) from the full cohort to serve as the subcohort. With sampling fractions of ≥0.10, results of a case-cohort analysis are similar to the full cohort analysis [13]. The cases together with the subcohort define the actual population under study. The advantage of this design is that it enables the performance of survival analyses without the need to score all 3559 chest CT scans.

### CT Scanning

Between January 2004 and December 2007 all subjects underwent a volumetric chest CT in full inspiration. CTs were obtained without cardiac or respiratory gating on 16-slice MDCT scanners with a collimation of 16×0.75 mm. The participants at UMCG were scanned on a Sensation-16 CT (Siemens Medical Solutions, Forchheim, Germany), whereas the participants at UMCU were scanned on either a Mx8000 or Brilliance-16P CT (Philips Medical Systems, Cleveland, OH, USA). Exposure settings were adjusted according to body weight: 80–100 kVp (<50 kg), 120 kVp (50–80 kg) or 140 kVp (80 kg or more) at 30 mAs, yielding a computed tomography dose index of 0.8, 1.6 and 3.2 mGy, respectively. Axial images with a slice thickness of 1-mm at 0.7-mm increment were reconstructed using a smooth reconstruction filter (Siemens B30f, Philips B-filter).

### Scoring of CT Characteristics

CT scoring was performed at a research workstation (iX Viewer; Image Sciences Institute). Left anterior descending coronary artery calcification (CAC) and descending aortic calcifications (DAC), were scored as previously described [14], [15]. Briefly, calcifications in the left anterior descending (LAD) were assessed using the following scale; grade 1, mild (1–2 focal [limited to < = 2 slices] calcifications); grade 2, moderate (>2 focal calcifications or a single calcification extending for >2 slices); and grade 3, severe (fully calcified coronary arteries extending over multiple segments). The lower margin of the descending aorta was defined as the level from where the diaphragm could be seen. The number and size of aortic wall calcifications were assessed and graded as follows: grade 0, absent; grade 1, mild (< = 3 focal calcifications); grade 2, moderate (4–5 focal calcifications or 1 calcification extending for > = 3 slices); and grade 3, severe (>5 focal calcifications or >1 calcification extending for > = 3 slices).

CT scoring was performed by a research physician with two years of experience in reading chest CT. The reader was blinded for participant’s characteristics and outcome status. Adequate scoring of the chest CT was assessed by evaluating the reproducibility of the visual grading between the research physician and an experienced board certified chest radiologist for a subset of 50 randomly selected chest CTs, that were part of this study. Weighted kappa’s were 0.85 for CAC and 0.72 for DAC, reflecting a good interobserver agreement between the research physician and the chest radiologist.

### Follow up and Cardiovascular Events

Data on fatal and non-fatal CVD events were obtained from the Dutch National Registry of Hospital Discharge Diagnoses and the National Death Registry from baseline to January 2008. According to the International Classification of Diseases (ICD) the Dutch National Registry codes all discharge diagnoses as ICD-9 and the causes of death as ICD-10 codes [16]. The database was linked to the study cohort with a validated probabilistic method [17], [18].

Using the ICD-9 codes, we categorized cardiovascular disease events (codes 390 to 459) as coronary heart disease (CHD) (codes 410 to 414), including acute myocardial infarction (AMI) (code 410), cerebrovascular disease (codes 430–438) or other cardiovascular disease.

Using the ICD-10 codes, we categorized cardiovascular deaths (codes I00–I99) as ischemic heart deaths (codes I20–I25), cerebrovascular death (codes I60–I69) or death due to other cardiovascular disease.

Whenever multiple events occurred, the first diagnosis was taken as an end point, aside from cardiovascular deaths, which prevailed over hospital admissions.

### Statistical Analyses

The baseline characteristics of the CVD cases (n = 263) and the subcohort (n = 341) are presented. Medians and quartile limits (quartile 1 to 3 [Q1–Q3]) were computed for the continuous variables, as they all showed skewed distributions. Categorical variables were expressed as frequencies. The numbers of subject with moderate DAC were small, therefore we grouped moderate and severe together. Similarly, moderate or severe CAC were grouped together.

CVD event rates stratified according to smoking status were estimated, by dividing the number of CVD events in the corresponding smoking status category by the number of person-years at risk in that category.

To assess the relation between current smoking status and CVD events hazard ratios (HRs) and 95% confidence intervals (95% CIs) for CVD events were calculated for current smokers, with former smokers as the reference group. We used a Cox proportional hazards model with an estimation procedure adapted for the case-cohort designs. These adaptations were carried out with the method according to Prentice in which all subcohort members are equally weighted [12]. Age (continuous), pack years (continuous), CAC (categorical) and DAC (categorical) were evaluated for confounding the association of smoking status and CVD risk.

Additionally, we performed tests for effect modification by CAC and DAC by including multiplicative interaction terms with these variables and smoking status. Analyses were performed with R-project software package, version 2.15 (www.r-project.org).

## Results

During a median follow up of 2.6 years (Q1–Q3, 1.5 to 3.1), 263 CVD events occurred among the 3559 subjects of the baseline cohort (Table 1), 18 CVD events were fatal and 245 were non-fatal. The median age subjects experienced a fatal CVD event was 63.3 years and the median age for experiencing a non-fatal CVD event was 63.1 years. The majority of all CVD events involved CHD events (54%).

**Table 1:** Specification of the 263 cardiovascular events recorded over a median follow up time of 2.6 year.

Type of cardiovascular event	ICD-9/ICD-10 codes	Number of events
**Non- fatal cardiovascular disease events**		**245**
Coronary heart disease	410–414	137 (56%)
Cerebrovascular disease	430–438	36 (15%)
Other cardiovascular disease	401–405, 420–429, 440–449	72 (29%)
**Fatal cardiovascular disease events**		**18**
Coronary heart disease	I20–I25	7 (39%)
Cerebrovascular disease	I60–I69	3 (17%)
Other cardiovascular disease	I30–I52, I70–I79, R00–R09	8 (44%)

Table 2 presents the baseline characteristics of the CVD cases and the subcohort. As expected, the cases were slightly older, were more often current smokers and had more numerous and more severe cardiovascular calcifications compared to the subcohort. The number of pack years smoked were comparable among both groups.

**Table 2:** Baseline characteristics of cardiovascular disease cases and the subcohort.

	Cases	Subcohort
	n = 263	n = 341
**Age, years**	61.4 (57.4–65.8)	60.3 (56.5–64.0)
**Pack years, years**	38.7 (29.7–49.5)	38.7 (28.0–49.5)
**Years of smoking cessation, years**	6 (3–10)	5 (2–8)
**LAD** * **coronary artery calcifications (%)**		
absent	16	37
mild	15	18
moderate/severe	69	45
**Descending aorta calcifications (%)**		
absent	39	62
mild	21	17
moderate/severe	40	22
**Smoking status (%)**		
current smoker	63	58
former smoker	37	42
**Follow up time, years**	1.4 (0.7–2.1)	2.9 (2.7–3.3)

Values of continuous variables are expressed as median (range) because of non-normal distribution. Categorical variables are expressed as percentages.

* LAD, Left Anterior Descending.

The mean annualized event rate 18.65 events/1000 person years (95% CI 15.11–22.78) for former smokers versus 26.88 events/1000 person years (95% CI 22.95–31.28) in current smokers.

In Table 3, unadjusted and adjusted HRs and the 95% CIs for CVD events are presented for current smokers compared with former smokers. The unadjusted HR of 1.31 (95% CI 1.03-1.68) as well as the for age, pack years and cardiovascular calcifications (CAC and DAC) adjusted HR of 1.33 (95% CI 1.00–1.77) indicate that there was a statistically significant positive association between current smoking status and CVD events.

**Table 3:** CVD event HRs and 95% CIs in current smokers as compared with former smokers.

	HR (95% CI) for current smoker
Crude	1.31 (1.03–1.68)
*Age and pack years adjusted	1.48 (1.12–1.97)
†Age, pack years, CAC ‡ and DAC§ calcification adjusted	1.33 (1.00–1.77)

* Adjusted for age (continuous) and pack years (continuous).

† Adjusted for age (continuous), pack years (continuous), Left Anterior Descending.

‡ Coronary Artery Calcifications(3 categories),

§ Descending Aorta Calcifications (3 categories).

In additional analyses that incorporated multiplicative interaction terms, neither CAC nor DAC modified the association between smoking status and CVD risk (P = 0.08).

## Discussion

In this case cohort study, comprising a population of male subjects with a long and intense smoking history, followed for a median period of 2.6 years, we were able to demonstrate that current smoking behavior was associated with a 31% greater risk of CVD events compared to former smoking status. This positive relation remained significant after adjustment for age, number of pack years and different types of cardiovascular calcifications. The effect of smoking continuation on CVD risk is identical for all grades of coronary and aortic calcifications (i.e., no interaction or effect modification).

### Smoking as CVD Risk Factor in Heavy Smokers

Our findings are consistent with previous studies demonstrating an increased CVD risk among current smokers compared to never smokers and former smokers [19]–[21]. Since our study was conducted in a population comprising subjects with a long and intense smoking history, our results underline that current smokers remain to have an increased CVD risk compared to ex-smokers despite the fact that both groups have a heavy smoking history of on average 40 pack years. An explanation for this risk difference could be that smoking increases CVD risk due to multiple mechanistic pathways with differing temporal responses to smoking cessation [19]–[21]. So, on one hand, smoking can causes irreversible damage, where increased exposure to smoking leads to more damage and increases CVD risk [9], [22]. Smoking has, on the other hand, potential reversible effects like platelet activation, coronary spasm and ventricular arrhythmias attributing to the CVD risk difference between current en ex-smokers [5], [20].

### Smoking as CVD risk Factor Independent of Cardiovascular Calcifications

We demonstrated that the effect of current smoking status on CVD risk is independent of coronary and aortic calcifications. This finding adds to prior research demonstrating that the absence of CAC might not be as reassuring in those who smoke, since smokers without CAC have an increased relative risk of CVD events and all-cause mortality [23], [24]. Factors associated with an increased relative risk of CVD events in smokers without cardiovascular calcifications might partly be attributed to the potential presence of non-calcified plaques, which might be more prone to rupture than calcified plaques [25]. Furthermore, smoking causes inflammation of these vulnerable non-calcified plaques [20].

Smoking as well as aortic calcifications have been proposed to cause an increased CVD risk by the common underlying process of atherosclerosis [26]. However, we demonstrated that the effect of current smoking status on CVD risk when jointly modeled with DAC did not attenuate. This implies that smoking also causes an increased CVD risk via other mechanisms than the with aortic calcifications shared atherosclerotic pathway.

Concisely current smoking status exerts its hazardous effects on CVD risk by pathways that differ from the mechanisms by which smoking causes cardiovascular calcifications. These findings support the irreplaceable role of smoking status, for CVD risk evaluation.

### Effect Modification of Smoking on CVD Risk by Cardiovascular Calcifications

Our study as well as previous studies have demonstrated that smoking as well as coronary and aortic calcifications are considered to be independent risk factors for CVD, enhancing the possibility for cardiovascular calcifications to magnify the adverse effects of current smoking on CVD risk [26]–[28]. The present data did not provide evidence for a potential interaction between current smoking status with coronary and aortic calcifications. However, in our results the formal test for interaction was of borderline significance (P = 0.08), making further investigation in cardiovascular calcifications subgroups worthwhile.

### Remarks

We used a simple and accurate semi-quantitative assessment [14] for grading the CAC and DAC on ungated low-dose CT images. Using low dose scans gives rise to the possibility to miss extent of small lesions. However, as small calcifications are more common in those younger than 50 years [29] and as our study population comprised subjects aged 50 years or older, we do not expect this phenomenon to have affected our results.

Furthermore, CT scans were performed on 16-slice scanners with inferior spatial and temporal resolution and subsequently less accurate assessment of calcifications compared to 64-slice [30]. Similarly, usage of quantitative volume measurements instead of semi-quantitative assessments gives a more precise quantification of the cardiovascular calcifications. Though, if this would lead to a better prediction of the attenuation of the CVD risk estimates by cardiovascular calcifications is debatable. Ongoing studies are investigating the replacement value of automatic quantitative measurements of the coronary arteries and aorta calcifications by semi-quantitative assessments.

In addition, we have limited the visual grading to calcifications in the left anterior descending coronary artery and descending aorta. So we cannot be sure if current smoking exerts its hazardous effect on CVD risk independent of calcifications in the whole coronary tree or aorta. Although, we can expect this relation to be true as the CAC distribution in the coronary tree reflects the natural history of the disease, starting at the first 2 cm of the left anterior descending coronary artery, followed by the right coronary artery, left main and left circumflex coronary artery (LCX) [31] thereby verifying previous pathological anatomic studies [32] and analysis of coronary angiography [33]. It has also been demonstrated that calcifications in the descending aorta, in particular, are more dominant in all CVD events compared with ascending aorta calcifications and both ascending and descending aorta calcifications [8].

Moreover, the data on smoking status originates from self-completed questionnaires without biochemical verification of the smoking status, with the risk of social desirability response bias. However, self-reports on smoking behavior appeared to be valid in a lung cancer screening setting [34].

### Study Limitations

One of the shortcomings of this study is the limited generalizability of the outcomes because this study was conducted in a male lung cancer screening population of current or former smokers with a smoking history >16.5 pack years. We cannot be sure that current smokers with a smoking history <16.5 pack years, female gender or individuals who receive a chest-CT for other reasons than lung cancer screening have an increased CVD risk of 33% compared to ex-smokers.

Another potential weakness is that smoking status is determined at baseline and treated as a time-independent factor, i.e. not changing in time. Dichotomizing smoking status can be suboptimal since it is likely that someone who stopped smoking at baseline and restarts smoking during follow up (i.e., mixed smoking history) is suspected to be at a higher CVD risk than a person who remains a quitter. This nondifferential misclassification that could arise over time may dilute the difference in CVD risk between ex and current smokers, resulting in an underestimation of the true HR. However, a review on the magnitude of risk reduction achieved by smoking cessation in patients with CHD showed that the risk reduction reported in studies excluding patients who reported mixed smoking histories did not statistically differ from studies that did not account for this misclassification [35]. Furthermore, collecting reliable data on smoking is challenging and many prognostic models like the Framingham [36] and PROCAM [37] risk score have been developed with smoking as a time independent factor.

Additionally in observational research, like in our case cohort study, unobserved confounding could be a source of bias [38]. Since good life style behavior appear to cluster, e.g. persons who quit smoking appear to have higher rates of diet and exercise modifications that effectively lower CVD [39], there is always the chance that the reduced CVD risk among ex-smokers we observed was due to unmeasured health attitudinal characteristics inherent to smoking cessation.

### Conclusions

Current smoking status remains an important CVD event risk factor even in a population of heavy smokers. Current smoking exerts its hazardous effects on CVD risk by pathways independent of cardiovascular calcifications. These findings support the irreplaceable role of smoking status, for CVD risk evaluation. Our data reinforce the notion that all current smokers, including those with a heavy smoking history and those with and without cardiovascular calcifications, should be encouraged to quit.
